# The Relationship between Sustainable Built Environment, Art Therapy and Therapeutic Design in Promoting Health and Well-Being

**DOI:** 10.3390/ijerph182010906

**Published:** 2021-10-17

**Authors:** Zhen Liu, Zulan Yang, Mohamed Osmani

**Affiliations:** 1 School of Design, South China University of Technology, Guangzhou 510006, China; 2 School of Architecture, Building and Civil Engineering, Loughborough University, Loughborough LE11 3TU, UK; m.osmani@lboro.ac.uk

**Keywords:** health, therapeutic design, built environment, art therapy, sustainable development, digital technology, building information modeling (BIM), bibliometric analysis, VOSviewer

## Abstract

At present, a smart city from the perspective of the United Nations Sustainable Development Goals (SDGs) emphasizes the importance of providing citizens with promising health and well-being. However, with the continuous impact of coronavirus disease 2019 (COVID-19) and the increase of city population, the health of citizens is facing new challenges. Therefore, this paper aims to assess the relationship between building, environment, landscape design, art therapy (AT), and therapeutic design (TD) in promoting health within the context of sustainable development. It also summarizes the existing applied research areas and potential value of TD that informs future research. This paper adopts the macro-quantitative and micro-qualitative research methods of bibliometric analysis. The results show that: the built environment and AT are related to sustainable development, and closely associated with health and well-being; the application of TD in the environment, architecture, space, and landscape fields promotes the realization of SDGs and lays the foundation for integrating digital technologies such as Building Information Modeling (BIM) into the design process to potentially solve the challenges of TD; and the principle of TD can consider design elements and characteristics from based on people’s health needs to better promote human health and well-being.

## 1. Introduction

One of the important goals of smart cities is to provide citizens with a good living environment and improve their overall quality of life [1]. The British Standards Institute defines the smart city as “the effective integration of physical-digital systems and human systems in the built environment to provide citizens with a sustainable, prosperous and inclusive future” [2]. Health and well-being quality of life assessment indicators can be used as a measure of the sustainability of the community [3,4]. The United Nations Sustainable Development Goal (SDG) 3 also proposes to promote the well-being of all people of all ages and ensure a healthy lifestyle [5]. Integrating urban buildings with related technologies such as sensing, communications, and cloud computing to collect real-time information is an indispensable aspect of the efficient operation of smart cities [6]. Moreover, with the efficient operation of smart cities, there is potential to achieve many goals of the United Nations for sustainable development [7], such as SDG 11. SDG 11, which is to build inclusive, safe, resilient, and sustainable cities and human settlements, proposes to build sustainable and disaster-resistant buildings [8]. However, the world is facing an unprecedented global health crisis of the current coronavirus disease 2019 (COVID-19), which has affected the health of more and more people. World Health Organization (WHO) puts forward a number of anti-epidemic suggestions, such as physical distancing, wearing a mask, and avoiding crowds [9]. However, long-term social isolation will lead to the decline of memory cognitive ability, and learning ability [10]. Moreover, for people with relatively vulnerable psychological conditions, such as young people (18–29 years old), disadvantaged groups, or individuals with previous mental health problems, social isolation will have a more serious impact on their mental health [11]. In addition, when implementing epidemic prevention measures for staying at home for a long time, Amerio et al. [12] consider that the built environment of housing is one of the factors affecting people’s mental health, and the impact of the built environment on mental health also needs to be investigated from multiple disciplines, such as urban planning and environmental health.

Since sustainable development is designated to benefit everyone, urban development plays a key role, where the continuous increase in urban population poses challenges to people’s health [13]. Moreover, the ongoing impact of the COVID-19 has a negative effect on the mental health of various groups of people [14], such as anxiety, depression, and the disturbance of emotions, sleep, appetite, and sociality [15]. Among the various treatments that promote human health, art therapy (AT) has been gradually used for decades. AT is a form of expressive therapy that employs creative art processes [16] to enhance peoples’ mental health [17], well-being [18], and quality of life [19]. It can provide a medium for the emotional expression of the treated person, and can also be used as a channel for emotional release and self-expression, so as to achieve a satisfactory therapeutic effect [20]. Furthermore, AT is increasingly accepted in the health discipline, especially in the field of mental health, because it is deemed as an effective way to actively deal with the variables of mental disorders, personal and family conflicts, and enable patients to experience their own difficulties, conflicts, and fears in a less painful way [21]. Daykin et al. [22] believe that art, design, and the environment have a positive impact on mental health, and proposed that the impact of architectural design on mental health is very important, particularly for people with dementia. By viewing the relationship between art and design, measures to promote health can be summarized as therapeutic design (TD) from the perspective of design. TD is to reduce stress and promote healing through aesthetic enhancement [23]. However, although an increasing number of studies support the mutual promotion of TD and health, few studies analyze effective interventions to promote health from a design perspective in the context of sustainable development, and summarize what specific research is available on TD. Therefore, this paper aims to assess the relationship between building, environment, landscape design, AT, and TD in promoting health within the context of sustainable development.

## 2. Methods

Bibliometric analysis was adopted to address the aim of the paper. It analyzes the keywords, chronological trend, research methods, research subjects, and research topics of the included literature from a quantitative and qualitative perspective. The principle of bibliometrics is to analyze scientific or technical activities through quantitative research on publications and to understand the research dynamics of science and technology with quantitative indicators [24]. The method of bibliometric analysis can draw general conclusions on research results and the impact on specific knowledge areas without a thorough bibliographic review [25]. Further, the research methods of bibliometric analysis can objectively and directly display the characteristics and laws of existing research, so as to provide reference and inspiration for the next stage of research [26]. No attempts were previously made to use bibliometric analysis to explore the research content and potential connections of the four themes of the built environment, AT, TD, and sustainable development. This paper set out to use the bibliometric analysis to assess potential connections between building, environment, landscape design, AT, and TD in the context of sustainable development from both macro-quantitative and micro-qualitative aspects.

In the bibliometric analysis, this paper imports the results of bibliometric research into the VOSviewer software to generate bibliometric maps for macro-quantitative keyword visualization analysis and then uses micro-qualitative analysis to review the content of related articles. The VOSviewer software is adopted for keyword analysis, since it is not only a visualization tool through language algorithms and text mining technology but also a commonly used tool in the scientific quantitative analysis [27]. The bibliometric map produced by visualization tools is a quantitative analysis method that can describe the structure and development process of publications in different fields [28]. In addition, keywords are an important part of the literature to reflect the core content, and the analysis method of keyword co-occurrence can well reflect the current academic research hot spots, knowledge structure, and development trend of some disciplines [29,30]. Moreover, co-occurrence analysis is a data analysis method combining quantitative and qualitative analysis, which can perform cluster analysis according to the frequency of keywords appearing in the paper [31]. Therefore, this paper uses the visual keywords of the VOSviewer software to reveal and verify the relationship between sustainable development, building, environment, landscape design, AT, health and design, and then lays a theoretical foundation for the research summary of TD.

**Figure 1:**
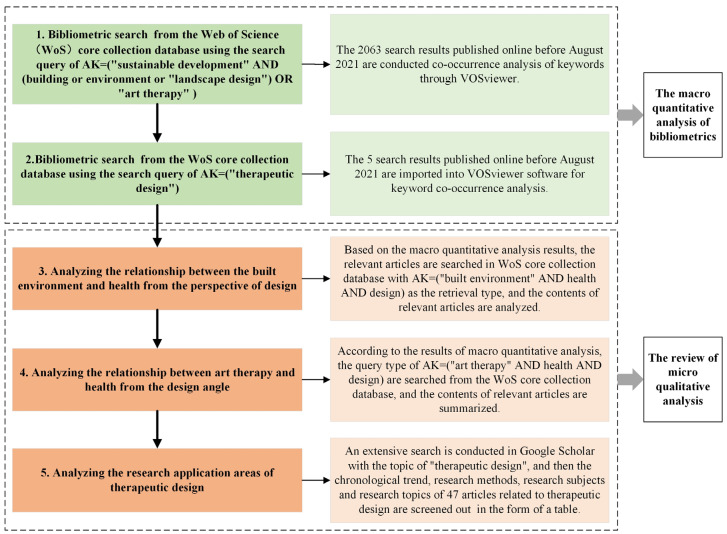
The flow chart of the research methodology.

The flow chart of the research methodology of the paper is shown in Figure 1, in which the investigation and research are carried out in five stages through quantitative and qualitative analysis processes: (1) bibliometric search of published articles related to building, environment, landscape design and AT in the context of sustainable development for further conducting co-occurrence analysis of keywords through VOSviewer; (2) bibliometrics search of the articles with TD as the keyword for co-occurrence analysis of keywords via VOSviewer; (3) analyzing the relationship between the built environment and health from the perspective of design; (4) analyzing the relationship between AT and health from the design angle; (5) analyzing the research application areas of TD. Among these five stages, the first two stages are the macro-quantitative analysis of bibliometrics, and the latter three stages indicate the summary content of micro-qualitative analysis. When searching related articles with TD as a keyword in the Web of Science (WoS) core collection database, there are few articles related to the topic. Therefore, in order to add the most relevant research content of TD in the process of qualitative analysis, this paper takes TD as the theme and conducts an extensive search of academic literature in Google Scholar. Then, the research content of TD is summarized through the micro-qualitative analysis of bibliometrics.

## 3. Results

### 3.1. Results of the Macro Quantitative Analysis of Bibliometrics

#### 3.1.1. Keywords Analysis of Building, Environment, Landscape Design and Art Therapy (AT) in the Context of Sustainable Development

**Figure 2:**
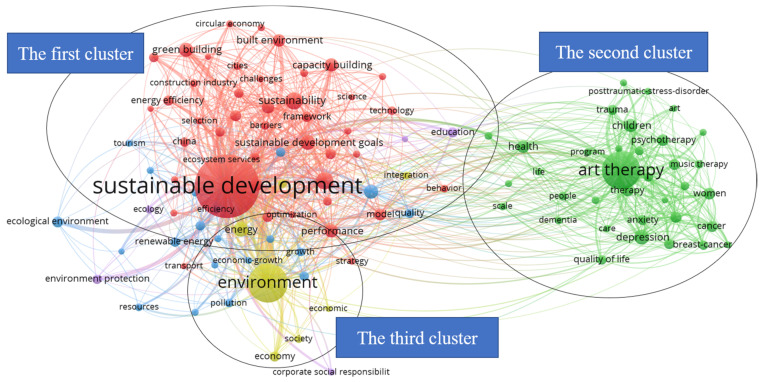
VOSviewer keyword network visualization map of building, environment, landscape design and art therapy in the context of sustainable development within the Web of Science (WoS) core collection database (devised by the authors).

Figure 2 is the VOSviewer keyword network visualization map of the building, environment, landscape design, and AT in the context of sustainable development. In the analysis map of network visualization generated by VOSviewer in Figure 2, the size of the circle indicates the frequency of each keyword. The larger the circle, the more frequently the keyword appears; and the closer the distance between the two circles, the closer the connection between the two. In addition, the thickness of the lines of the links between the different circles indicates the strength of the keywords. The more lines there are, the more the keywords have a co-occurrence relationship with the keywords. Moreover, circular nodes use different colors to represent different clusters. As shown in Figure 2, these keywords are grouped into three clusters in terms of the color and size of the circular node, of which the first cluster is based on the red keywords of sustainable development; the second cluster is focused on the green keywords of AT, and the third cluster is related to the yellow keywords of environment. In terms of the number of linked lines, Figure 2 shows that sustainable development and AT have a co-occurring relationship with a number of keywords. In assessing the keywords of sustainable development, environment, and AT among the three clusters, the common keyword that is most closely related to them is health.

**Figure 3:**
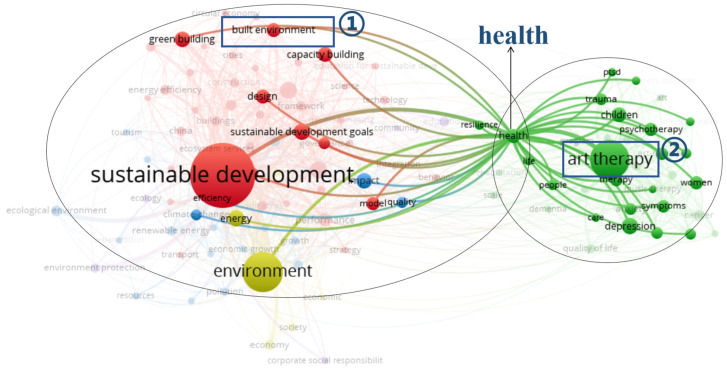
Network visualization map with the theme of health in the WoS core collection database via VOSviewer (devised by the authors).

**Figure 4:**
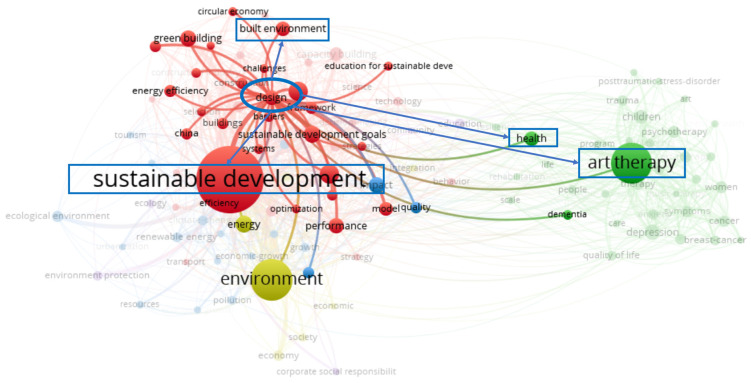
Network visualization map with the theme of design in the WoS core collection database via VOSviewer (devised by the authors).

As shown in the health-themed network visualization in Figure 3, the keywords of the three clusters can be further subdivided into two categories. The first category, which integrates the relationship between sustainable development and environment, and excludes keywords that are irrelevant to the research topic, such as model, climate change, energy, impact, quality, government, and efficiency, can be grouped under the theme of built environment and health. The keywords of the built environment and sustainable development in the first category have a co-occurrence relationship. The second category encompasses the themes of AT and health. In addition, as shown in Figure 4, in the network visualization diagram with design as the central theme, it can be seen that the theme of design has a co-occurrence relationship with health, AT, sustainable development, and the built environment. In general, the macro-quantitative analysis of bibliometrics has initially revealed the co-occurrence relationship between the keywords of sustainable development, built environment, and AT, as well as the close connection between the built environment and AT in the keyword of health. Further, the objective analysis of the VOSviewer software confirms that there is a potential relationship between TD for health promotion from a design perspective, AT, built environment, and sustainable development.

#### 3.1.2. Keyword Analysis of Therapeutic Design (TD)

**Figure 5:**
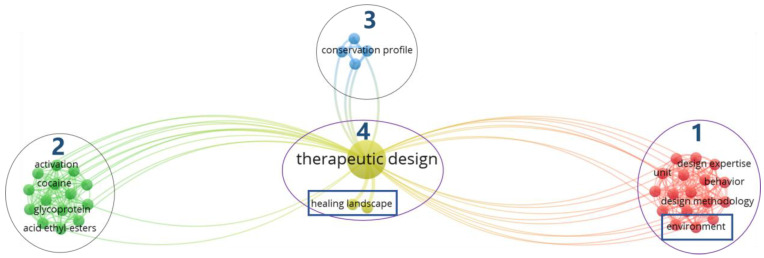
Network visualization map with the theme of therapeutic design (TD) in the WoS core collection database via VOSviewer (devised by the authors).

As shown in Figure 5, VOSviewer divides the keywords of the articles with TD as keywords in the WoS core collection database into four major clusters, such as red, green, blue, and yellow. Excluding irrelevant keywords of biology and other professional medicine, the clusters related to the topic of this research are mainly in yellow and red. In each cluster, TD is closely associated with the keyword of healing landscape in the yellow cluster. In the red cluster, TD is closely related to the keyword of environment. In short, the research and application fields of TD are mainly related to landscape and environment. There are still relatively few applied articles on TD, which indicates that future research in this field has opportunities, such as the realistic needs of the COVID-19, and challenges, such as the innovation bottleneck under the health gap.

### 3.2. Results of the Micro Qualitative Analysis

A micro qualitative analysis is conducted based on the above results of VOSviewer’s visual keyword macro quantitative analysis. Firstly, the articles that have a close connection between the built environment and AT in promoting health are analyzed from a design perspective. Then, the relevant articles on TD are specifically described, analyzed, and classified from a wide range of search results. And finally, the chronological trend, research methods, research subjects, and the topics of TD are summarized from the categories of application fields.

#### 3.2.1. The Relationship between Built Environment, Health and Design

There is a mutually promoting relationship between the built environment and health, which mainly presents the design aspects of healthcare buildings and the application of Building Information Modeling (BIM) digital technology. A patient-centered built environment for sustainable healthcare is beneficial to the health and rehabilitation of patients [32]. When studying how the healthcare of the built environment affects human health, it is necessary to choose and apply the correct theory to establish an evidence base for the design of the built environment in order to develop more suitable health interventions [33]. In addition, in the design of outpatient healthcare buildings, the concept of health promotion includes three perspectives on health behavior, health equity, and sense of coherence [34]. When designing for the elderly, lighting quality, sound and smell are important Environmental Indoor Quality (IEQ) characteristics that promote the active life of dementia patients [35]. In addition, since the external environment and healthcare buildings have a significant impact on the mobility, independence, and quality of life of the elderly, the general design method should be considered when carrying out architectural design, and the design process should evaluate the problems related to the built environment and accessibility in an objective and self-reported manner [36]. In addition to considering the source of environmental satisfaction from the visual sense, environmental design has the potential to the sense of hearing and smell helps to reduce the pressure on the patient’s peers in children’s services [37]. In general, the design of the built environment has a positive effect on improving human health, which helps to achieve the good health and well-being proposed in SDG 3. However, the design of the built environment is mainly used in the healthcare field for the elderly, and applications in other fields need to be further expanded the scope of research for better alleviating the impact of the COVID-19 epidemic on people’s health.

In addition to the built environment can promote human health, the safety of the building itself also involves health issues. In this regard, BIM digital technology plays an important role. When faced with the safety problems of construction and incomplete design information, combining the building safety ontology, that is, the building safety management knowledge, with BIM digital technology can make the security knowledge query of the whole building ontology more efficient and realize the risk analysis and automatic security planning of BIM [38]. As a computer-aided design paradigm, BIM is used as a tool to diagnose and correct the defects of building structure design, so as to bring benefits to the whole process of building design, such as safety, cost improvement, time-saving, quality improvement, project management, and implementation [39]. In addition, the combination of the numerical model and BIM can enable designers to quickly control air quality during the life cycle stage of architectural design, which reflects a new paradigm of environmental sustainability from the concept of “healthy building” [40]. In addition, BIM that creates and manages the building information process is an essential catalyst of smart cities as it promotes the sustainable development of the city by improving the energy and cost-effectiveness of buildings [41], which has potential value for the realization of SDGs. However, there are still gaps in research on how BIM digital technologies can achieve the SDGs. Overall, BIM digital technology as an auxiliary tool can promote attention to health issues and improve the sustainability of architectural design throughout the life cycle.

#### 3.2.2. The Relationship between Art Therapy, Health and Design

At the design level, AT plays a vital role in promoting the health of patients, and art materials are the main research medium of AT. First, art is used in the field of mental health in various ways, among which AT is an effective method that is applicable to many groups in the field of health care [42]. AT explores the patient’s inner world by using non-threatening therapeutic relationships and art materials to improve the patient’s illness [43]. AT can not only promote mental health but also reduce mental illness [44]. The intervention of AT can strengthen the self-function of adult health [45]. Lee et al. [46] believe that integrating the ecological environment into art psychotherapy can play a vital role in improving mental health, such as stress, depression, anxiety, and aggression. Bonsai can be used as a therapeutic material to promote AT, which can overcome trauma in psychodynamic therapy interventions and maintain good mental health for patients [47]. AT provides a model that not only treats art materials as a language to create expression space, but also uses art production as a valuable form of expression, and then maintains it in a broader context expression to stimulate the process of collaborative design [48]. Further, in the practice of AT, understanding the behavioral participation of the elderly with cognitive impairments in the creation and sharing of artworks from the perspective of empathy and reflective design can enhance their social engagement [49]. In addition, art-based methods and aesthetics can develop passion and emotional connections in many fields such as healthcare and management, which can inject sustainable values and results into individual, organizational, and collective practices [50]. In general, the AT is an effective intervention to promote health, and the use of art presents a potential association with sustainability.

#### 3.2.3. Therapeutic Design (TD)

Both the built environment and AT could potentially have a positive effect on promoting health when they use the means of design for therapy. Based on the analysis results of the built environment and AT, this paper finds that TD is mainly divided into two areas of research application: the first one comprises therapeutic environment [51,52,53], architecture [54,55,56], and space [57,58,59]; whilst the second is a garden-based therapeutic landscape design [60,61,62]. The following sections carry out micro qualitative analysis from these two research application fields.

- Design of Therapeutic Environment, Architecture and Space.

The applied researches of TD in the fields of environment, architecture, and space are shown in Table 1. In terms of the chronological trend, the applied research of TD in the field of environment, architecture, and space occurred between 2010 and 2014. In terms of research method, the application research of TD in the fields of environment, architecture, and space mainly adopts literature review [23,63,64] and case study [65,66,67]. In terms of the research subject, the TD mainly focuses on the design of the nursing environment for patients with dementia to improve their quality of life [64,68,69], followed by the elderly [54,70,71] and children [57,58,72]. In terms of the research topic of TD, the research contents of environment, architecture, and space are interrelated. In addition, the TD oriented the environment, architecture, and space is mainly used in hospital places [23,55,65]. Further, in a therapeutic environment, it is mainly implemented for the physical environment [64,68,69] and the nursing environment [53,73,74]. The following section analyzes and describes the research content of TD in the fields of environment, architecture, and space.

The design of the healthcare environment is a kind of nursing tool of TD, which helps to promote people’s health. Gesler et al. [55] stress that the design of hospital spaces and buildings can be regarded as an effective ‘therapeutic environment’. The outdoor environment design of hospital buildings can have a positive impact on the rehabilitation process of patients [65]. The environment can be regarded as a nursing tool [66]. The collaborative design of researchers, intermediate consumers, such as customers and designers, and end-users, such as patients and their families, can form a healthcare environment that fosters healthier lives for people [51]. Osaji and Price [53] propose in a project studying the use of modeling, simulation, and visualization methods to improve TD of healthcare environments. The physical environment can promote health, happiness, and organizational productivity and profitability, so the design of psychosocially supportive design combined with the physical environment can better promote positive psychological emotions [75]. Further, the creation of a therapeutic environment through a humanistic building which refers to putting human well-being at the core of architectural design and environmental management can enhance and support the healthcare environment in the nursing and treatment process, and help promote the public’s mental health [52]. In short, environmental design is an effective care/therapy/recovery/rehabilitation tool, which has shown a huge positive effect in promoting human health and well-being. However, in the design process, the well-being of people should be the core, and different stakeholders should be considered.

When designing treatments for patients with dementia, the design of the therapeutic environment and architecture are effective interventions to promote health. Design as a treatment resource can promote the health and function of patients with dementia [73]. The design of the therapeutic environment is an important factor to maximize the function and quality of life of patients with dementia [67]. Design interventions in the built environment are beneficial to the treatment of patients with dementia in long-term care settings [76]. In the residential environment of dementia care, architecture has potential therapeutic effects. It can provide tools and opportunities to explore emotional and mental problems [56]. In addition, in the design of a dementia-friendly living environment, the focus should be shifted from the condition to the patient’s life experience, such as eating experience, personal enjoyment experience, and bedroom experience [74]. In the design of outdoor environments, familiarity, legibility, particularity, accessibility, comfort, and safety seem to have a significant impact on the health of patients with dementia [77]. Furthermore, the environmental intervention and housing design for patients with dementia, many aging-related issues, and environmental interventions for dementia have not been adequately addressed [71]. In general, environmental design is a potential therapeutic intervention for patients with dementia, which can bring good therapeutic effects to special user groups [78]. The design of therapeutic physical and social environments can also improve the quality of life of dementia patients in dementia care units [79]. In general, the design of the therapeutic environment is mainly based on dementia patients. These qualitative analysis contents can be extended to mitigate the impact of COVID-19 on people’s cognition and health.

When designing the nursing environment for the elderly, it is necessary to consider different environmental categories for TD and to evaluate the design results based on evidence. The treatment environment for older persons can be described as consisting of three interacting rather than interwoven categories, namely, the physical environment, people’s behavior and environment, and the organizational philosophy of care, in which a good treatment environment can support the well-being of older persons living in long-term care facilities [70]. Additionally, Barnes [54] summarizes the evidence on building layout, sensory environment, and residents’ privacy, and proposes that all evidence-based designs must reconsider compromise or dynamic in the form of post-occupancy assessment, and points out that older people have special environmental design needs, such as improving the quality of life. In addition, Grant and Sommers [80] also propose that the design of a nursing building environment should not only meet the basic needs of lighting, heating, and acoustics but also support the special behavioral needs of patients with Alzheimer’s disease. Generally speaking, people’s needs for the design of therapeutic architecture and environment include basic physiological needs and deep-seated emotional needs. From the perspective of health and well-being, people’s physical and psychological needs can guide the design elements and characteristics of the TD.

TD with children as the research subject is mainly aimed at therapeutic space design. Spatial design is very important for mental and behavioral health, and it will affect patients’ treatment investment, aggressive behavior, emotional state, service results, experience, and perceptions of the quality of care received [59]. Regarding the design of children’s treatment spaces, clinical concepts can be transformed into architectural and interior design methods, and the multi-level nature of the environment can be considered to promote and support clinical goals [57]. Seo and Cho [58] believe that in the TD of the atrium space of the children’s hospital, location, direction, passage, natural light, outdoor landscape, and game facilities are important environmental design elements that determine the success of TD. In addition, the principle of biophilic design which refers to the design of people as biological organisms with respect for the mind-body system as an indicator of health and well-being is also an important element to improve human health [81]. When integrating biophilic design principles and TD elements in outdoor spaces, children’s health and well-being can be improved [72]. However, the data on the impact of the psychiatric ward as a therapeutic space on the therapeutic effect is not clear, and more comprehensive methods are needed for further research [63]. In short, although the therapeutic space design has a good promotion effect on children’s health and well-being, there is still insufficient research on the therapeutic effect of the space design on psychosis.

- Therapeutic Landscape Design.

The applied researches of TD in the field of garden-based therapeutic landscape design are shown in Table 2. The related research progressively appeared from 2002 to 2018. In terms of the research method, the research of TD in the garden-based landscape design field mainly adopts a case study [61,82,83] and a mixed research method based on literature review and case study [84,85,86]. In terms of the research subject, the studies mainly take the general public as the research group to analyze the therapeutic characteristics and effects of landscape design. In the field of therapeutic landscape design, gardens are the main research area of TD [60,84,87]. The following section analyzes and describes the specific research content of TD in the field of garden-based landscape design.

The therapeutic landscape can integrate different design elements to bring positive effects to patients, and its integration with medical facilities has a key role in promoting health. A therapeutic landscape refers to a green public space that is beneficial to people’s physical health, mental health, and social health, as well as a therapeutic activity space that provides people with meditation, alleviates stress, and encourages social interaction [88]. When the landscape design is aimed at rehabilitation and curativeness, creating the natural foundation for patients, such as green plants, can bring positive changes to patients and provide employees with a relaxing space [61]. In addition, daylighting is an important factor that promotes health in the design of therapeutic landscape [89]. However, in the research of a therapeutic landscape design based on physical, social, and symbolic spaces, patients feel respected and empowered [90]. Since the landscape and architectural features of the green environment play a key role in patients’ sensorimotor and cognitive stimulation, the combination of landscape design and neuroscience can be used as a non-pharmacological treatment to alleviate Alzheimer’s disease [85]. Hence, the therapeutic landscape design is particularly suitable for medical facilities, where the design of attractive landscapes in the hospital environment is important for patient rehabilitation and health [59]. In the landscape design of hospitals, the cooperation of landscape architects and medical professionals can influence and create an environment that promotes all aspects of rehabilitation [83].

Based on the benefits of therapeutic landscape design, horticultural therapy also has potential value in promoting health. Horticultural therapy refers to the conscious use of plants and gardens for treatment and rehabilitation activities to promote personal health [91]. In horticultural therapy, a therapeutic garden incorporating natural elements is a sustainable form of landscape design, which can serve as a catalyst to promote public health and well-being. As garden design has taken on new meaning, landscape design has gradually begun to emerge in the fields of ecological restoration, decorative applications, environmental art, and therapeutic landscapes/gardens [92]. The American Horticultural Therapy Association defines a therapeutic garden as “a plant-led environment designed to promote interaction with nature’s healing elements” [84]. Garden designs with themes of healing, meditation, contemplation, and restorative all play a positive role in healthcare [93]. “Healing Garden” is a sustainable landscape form in the field of landscape design [60]. Its benefits are mainly reflected in the areas of cognitive, psychological, social, and/or physical well-being [94]. Moreover, hard landscapes and soft landscapes, such as gardens and parks, can be used as a way to balance the lives of urban residents, increase their comfort level, and reduce stress [95]. In addition, therapeutic gardens incorporating natural elements in therapeutic landscape design have a certain potential to improve the mental and physical health of individuals [96]. Moreover, the use of aromatic plants and building materials in the garden design can bring good care effects for the elderly [97]. However, when investigating the public’s perception of the importance of horticultural therapy, Lakić et al. [98] find that the public is not sufficiently aware of the benefits of horticultural therapy. In general, landscape space design can improve the health and well-being of users, among which landscape design using the healing power of nature can aid the recovery and healing process of trauma patients [86]. Garden-based therapeutic landscape design can improve the mental and physical health of individuals, and confirms the important role of the environment in a person’s overall health and well-being [96]. In general, both therapeutic landscape design and therapeutic garden design make full use of the power of nature to promote health, which provides inspiration for the realistic needs of the COVID-19 epidemic.

## 4. Discussion

### 4.1. TD and Sustainable Development

Based on the results of Section 3.2.1 and Section 3.2.2, the research fields that realize therapy by design means mainly include the built environment and AT. In addition, the keyword network visualization map in the Section 3.1.1 indicates that both the built environment and AT have a certain connection with sustainable development. A review of related research finds that the development of the urban built environment has become a key determinant of the sustainability of the entire urban system [99]. Giles-Corti et al. [100] believe that decisions on how to design, construct, manage and govern cities will profoundly affect the health and well-being of urban residents, and the extent to which cities can achieve the United Nations SDGs. Moreover, the urban built environment is an important part of the urban ecological environment, which affects the quality of life of urban residents [101]. Furthermore, the construction of cities, buildings, and materials are potential means for achieving sustainable design [102]. Section 3.2.1 also proposes that the design of the built environment can contribute to the realization of SDGs from the health level and the application of digital technologies such as BIM. BIM can reduce construction waste in the whole life cycle of architectural design projects [103], which better promotes the realization of SDG 11 from the perspective of waste management of urban environmental impacts. Hence, the application characteristics of comprehensive TD in the field of the built environment ensures that TD promotes the health of citizens, and achieves SDGs. Besides the built environment, AT as a valuable expression can also bring better therapeutic effects from the design level. Applying art in different fields can inject sustainable value and results into organizational practices [50]. The art shows great hope in the direction of sustainable development [104]. Therefore, using art as the application medium of TD can not only maintain the patients’ good mental health but also has the potential to achieve sustainable development. In general, the analysis results in Section 3.2.1, Section 3.2.2, and Section 3.2.3 indicate that the application of TD in the field of the built environment and AT can not only promote human health but also have a potential connection with the realization of sustainable development.

### 4.2. TD and Digital Technology

The results in Section 3.2.3 indicate that different research subjects have different therapeutic needs and design characteristics. For example, patients with dementia should meet the design aspects of the therapeutic environment from the needs of nursing care, and children should meet the design issues of the therapeutic space from the needs of biophilia. From the perspective of health and well-being, people’s satisfaction with physical needs gradually develops toward psychological needs. In addition, the design features of therapy have gradually risen from the most basic design elements, such as light, smell, and direction to the design theme for exploring the patient’s inner emotions. In general, TD can consider design elements and characteristics according to people’s health needs, so as to better promote human health and well-being. However, the results in Section 3.2.3 highlight that although TD can promote human health through the effective space layout, architecture, environment, and landscape, it also faces four challenges in the design process: (1) conflict and change of design requirements [90]; (2) incomplete consideration of design features [71,73]; (3) lack of tools and framework for assessment [54]; (4) incomplete data collection [22,63,79].

In response to the above challenges, Section 3.2.1 reveals that the advantages of BIM digital technologies have potential effects in promoting health. Moreover, Section 3.2.3 points out that TD is mainly used in the fields of space, architecture, and landscape, which shows that TD and digital technology have a potential integration. In general, the results of Section 3.2.1 and Section 3.2.3 lay the foundation for the integration of BIM digital technologies into the TD process. In architectural design, BIM is a technology that uses three-dimensional models to establish a set of project databases, through the retrieval and application of information, to achieve the SDGs of the entire life cycle of the project [103]. Based on a sustainable and transparent BIM collaborative working environment, it has two benefits: (1) component information can be copied and verified in the environment so that all participants can communicate in time throughout the life cycle of the construction project, and (2) each participant can make scientific analysis through the design information shared by different design professionals to make corresponding decisions and better manage the project [105]. In addition, designers can use BIM for visual design, which can intuitively understand the space of key parts and arrange the corresponding components reasonably, so as to make better use of the limited space and effectively improve the design efficiency and quality [106]. In short, the use of BIM innovative technology can realize design communication, data exchange, and information sharing between collaborators, and improve collaboration between stakeholders [107]. Therefore, based on the advantages of BIM digital technologies, the integration of digital technologies into the TD process has the potential to solve the challenges in the TD process, so as to better promote human health and well-being.

## 5. Conclusions

This paper uses the method of bibliometric analysis to explore the built environment and AT for sustainable development via TD. The main contributions of this research are as follows: (1) In terms of research content, this paper is the first attempt to explore the relationship between AT, architecture, environment, landscape, health, and TD within the context of sustainable development through macro quantitative analysis and micro qualitative analysis of bibliometric. This paper reveals that the built environment, AT, and TD have the potential to promote public health and realize the sustainable development of the city. In addition, TD can consider design elements and features in line with people’s health needs. And the application areas of TD lay the foundation for the integration of digital technologies such as BIM into the design process to solve the challenges of TD. (2) In terms of research method, VOSviewer, a visual bibliometric map tool, is used to conduct keyword co-occurrence analysis, where the network visualization map of keywords provides an in-depth insight into the relevant topics of TD, providing a reliable research method for future research. Compared with the existing similar literature, this paper is the first to adopt the research method of keyword co-occurrence to incorporate relevant articles from multiple dimensions of TD. (3) In terms of the value of research, research on multiple dimensions of TD has certain research timeliness, practical and innovative significance for alleviating the impact of COVID-19 on society. The result of multi-dimensional TD research not only has a reference value for therapeutic designers, architects, and landscape architects, but also provides a potential development idea for information visualization software suppliers. Although the method of bibliometric analysis can scientifically reflect the research status and application fields of the subject content of the literature, due to the complexity of scientific development, the use of bibliometrics can only make a very rough measurement of the law of scientific development. However, the use of VOSviewer software for objective quantitative analysis in this paper can reduce the impact of this limitation and increase the rigor of the research content. As the search results of bibliometrics in a database are more reliable, the keyword analysis of VOSviewer in this paper only focuses on related articles in the WoS core collection database. Although in the context of sustainable development, the keyword connection between architecture, environment, landscape design, and AT only represents the database, it does not affect the classification of the research application field of TD and the specific content of micro-qualitative analysis. Future follow-up research could consider using different databases, such as Scopus, to extend the TD with the goal of promoting health to a wider range of digital technologies, such as BIM to Landscape Information Modeling (LIM) and City Information Modeling (CIM), and confirm the potential of digital technology in solving the challenges in the TD process through empirical research. In addition, future research could explore the practical applications of the built environment, AT, and TD in supporting sustainable development.

## Figures and Tables

**Table 1:** Research on the application of TD in the fields of environment, architecture and space.

Source	Year	Research Method	Research Subject	Research Topic
Cotton and Geraty	1984	Case study	Children	Therapeutic space design
Day et al.	2000	Literature review	People with dementia	Dementia care environment
Barnes	2002	Literature review	The older people	The design of caring environments: architectural design and physical environment
McCormick and Shepley	2003	Case study	General public	Therapeutic environment
Mitchell et al.	2003	Literature review and case study	Older adults with dementia	Outdoor environment
Gesler et al.	2004	Literature review and case study	General public	Hospital building program
Mazuch and Stephen	2005	Case study	General public	Healing environments: humanistic architecture
Chalfont	2006	Handbook	People with dementia	A therapeutic architecture for dementia care environments
Cioffi et al.	2007	Experimental observation	Residents with dementia, relatives and staff	The effect of environmental change
Daykin et al.	2008	Literature review	People with dementia and staff	The impact of art, design and environment in mental healthcare
Edvardsson	2008	Interview and observation	Older adults	Therapeutic environments
Landmark et al.	2009	Literature review	People with dementia	The physical environment
Davis et al.	2009	Literature review	People with dementia	Residential care environment
Van Hoof et al.	2010	Literature review and focus group	Older adults with dementia	Environmental Interventions and the design of homes
Chi et al.	2010	Case study	People with dementia	The therapeutic design of environments
Frazee	2010	Ethnography	Residents with dementia	Therapeutic physical and social environments in dementia care units
Osaji and Price	2010	Case study	General public	Healthcare environments
Nedučin et al.	2010	Case study	Patient	Hospital outdoor spaces
Habell	2012	Case study	People with dementia	Special environment
Pollock and Fuggle	2013	Case study	People with dementia	Therapeutic environment
Davidson	2013	Literature review and case study	Children	Therapeutic design elements in outdoor spaces
Papoulias et al.	2014	Literature review	Patient and staff	Therapeutic space
Chaudhury and Cooke	2014	Literature review and case study	People with dementia	The physical environment in dementia care settings
Marquardt et al.	2014	Literature review	People with dementia	The design of the built environment
Chaudhury et al.	2018	Literature review	Residents with dementia	The physical environment
Seo and Cho	2019	Literature review and interview	Children	Atrium space of children’s hospital
Wilson	2019	Literature review	Nurse	Hospital staff break areas
Liddicoat et al.	2020	Conceptual model	General public	The built environment of mental health services

**Table 2:** Research on the application of TD in the field of garden-based landscape.

Source	Year	Research Method	Research Subject	Research Topic
Riley	2002	Literature review	General public	Garden design
Meyerhoff	2002	Case study	General public	Therapeutic parking: gardens
Meyer	2007	Literature review and interview	The elderly	Scent gardens
Curtis et al.	2007	unstructured interviews and case study	Staff and service users	Therapeutic landscapes in hospital design
Joarder et al.	2010	Comparative experiment and statistical model	Patient	Daylight and outdoor views: therapeutic daylighting design
Gerlach-Spriggs and Healy	2010	Professional practice view	General public	The therapeutic garden
Bergeman	2012	Case study and recommendations	General public	Therapeutic landscapes
El Barmelgy	2013	Case study and framework	General public	Healing gardens’ design
Jiang	2014	Literature review	General public	Therapeutic landscapes and healing gardens
Lakić et al.	2015	Interview	General public	Horticultural therapy
Gonzalez and Kirkevold	2016	A cross-sectional E-Mail survey	The aged	Sensory gardens
De la Motte	2016	Case study	General public	Therapeutic garden designs
Belčáková et al.	2018	Case study	General public	Healing and therapeutic landscape: medical facilities
Abd Malik	2018	Case study and questionnaires	Park visitors (the disabled)	Park: therapeutic environment
McHugh and Ord	2018	Literature review and case study	General public	Therapeutic gardening
Zuanon and Cardoso de Faria	2018	Literature review and case study	Alzheimer’s disease	Landscape design and neuroscience cooperation
Czeck	2018	Case study	Landscape architects and medical professionals	Landscape design
Hartman	2020	Literature review, interview and case study	Veterans with post-traumatic stress disorder	Landscape space design
Iswoyo et al.	2020	Literature review and survey	Patient and landscape architects	Therapeutic landscapes in hospitals

## Data Availability

Publicly available datasets were analyzed in this study. This data can be found here: https://login.webofknowledge.com (accessed on 22 July 2021).
